# Extracellular Vesicles Produced by the Cardiac Microenvironment Carry Functional Enzymes to Produce Lipid Mediators In Situ

**DOI:** 10.3390/ijms24065866

**Published:** 2023-03-20

**Authors:** Varravaddheay Ong-Meang, Muriel Blanzat, Lesia Savchenko, Lucie Perquis, Mégane Guardia, Nathalie Pizzinat, Verena Poinsot

**Affiliations:** 1 Inserm, CNRS, Institut des Maladies Métaboliques et Cardiovasculaires U1964, Université Toulouse III—Paul Sabatier, BP 84225, CEDEX 4, F-31432 Toulouse, France; 2 CNRS, Laboratoire IMRCP UMR 5623, Université Toulouse III—Paul Sabatier, CEDEX 9, F-31062 Toulouse, France

**Keywords:** cargo, extracellular vesicles, inflammation, lipids, stromal cells

## Abstract

The impact of the polyunsaturated fatty acids (PUFAs) at physiological concentrations on the composition of eicosanoids transported within the extracellular vesicles (EVs) of rat bone marrow mesenchymal stem cells and cardiomyoblasts was reported by our group in 2020. The aim of this article was to extend this observation to cells from the cardiac microenvironment involved in the processes of inflammation, namely mouse J774 macrophages and rat heart mesenchymal stem cells cMSCs. Moreover, to enhance our capacity to understand the paracrine exchange between these orchestrators of cardiac inflammation, we investigated some machinery involved in the eicosanoid’s synthesis transported by the EVs produced by these cells (including the two formerly described cells: bone marrow mesenchymal stem cells BM-MSC and cardiomyoblasts H9c2). We analyzed the oxylipin and the enzymatic content of the EVs collected from cell cultures supplemented (or not) with PUFAs. We prove that large eicosanoid profiles are exported in the EVs by the cardiac microenvironment cells, but also that these EVs carry some critical and functional biosynthetic enzymes, allowing them to synthesize inflammation bioactive compounds by sensing their environment. Moreover, we demonstrate that these are functional. This observation reinforces the hypothesis that EVs are key factors in paracrine signaling, even in the absence of the parent cell. We also reveal a macrophage-specific behavior, as we observed a radical change in the lipid mediator profile when small EVs derived from J774 cells were exposed to PUFAs. To summarize, we prove that the EVs, due to the carried functional enzymes, can alone produce bioactive compounds, in the absence of the parent cell, by sensing their environment. This makes them potential circulating monitoring entities.

## 1. Introduction

Inflammation is a natural immune process, primarily involved in removing death tissues or pathogens. Cardiac repair upon myocardial infarction injury relies on coordinated immune cell infiltration to clean the infarct lesion, and foster angiogenesis and tissue remodeling [1,2]. After acute myocardial infraction (MI) and the activation of inflammatory pathways, neutrophils and monocytes infiltrate the damaged tissues within a few hours. However, monocyte infiltration is the only one maintained after days to promote reparative processes through angiogenesis and extracellular matrix deposition [3,4]. Throughout the reparation period, macrophages exhibit an almost anti-inflammatory phenotype [5] and secrete growth factors that recruit and activate mesenchymal cells, especially myofibroblasts and vascular cells, to shape the myocardial scar. The intensity and the duration of the inflammatory responses influence ventricular remodeling and clinical outcomes, particularly after myocardial infarction, where the continued existence of chronic low-level inflammation may lead to adverse remodeling [6,7]. Dysregulation of the inflammatory process, which is primarily orchestrated by the macrophages, may contribute to the development of heart failure through low grade chronic inflammation [8,9].

Preliminary publications reported that the surrounding medium from stem cells acts in a process of paracrine signaling, increasing cardiomyocytes’ in vivo and in vitro survival [10,11]. Later, extracellular vesicles (EVs), released by the mesenchyme stem cells into their surroundings, were supposed to be part of the paracrine signaling, sharpening the cardiac inflammation [12,13]. EVs, mainly derived from stem and progenitor cells, have been examined in trials as therapeutic solutions in the context of myocardial repair after MI [14,15]. Animals treated with such EVs usually present increased angiogenesis, reduced cardiomyocyte apoptosis and anti-inflammatory and immunomodulatory processes [16,17].

Recently, review articles reported about EVs obtained from different sources and considered their therapeutic potentials [18]. The therapeutic activity of EVs is principally attributed to their cargo (miRNA, DNA, proteins and lipids), which is reported as dependent on the parent cell type and state [19]. For example, MSC-EVs are almost all anti-inflammatory and polarize macrophages into M2 phenotypes [20,21]. In contrast, cardiosphere-derived cell EVs increase phagocytosis, efferocytosis and the M1 polarization of macrophages [22,23,24]. One must consider that besides the cell-to-cell communication mediated by the EVs, inter-kingdom EV communication also occurs between the host organism and the embedded guests [25].

microRNAs, carried by EVs, have especially been investigated as they can fine-tune cell function. Linked with these RNA, some proteins transported by these nanoparticles were also reported [26,27,28]. Only recently, interest was raised about EV lipid composition, mainly focused on the membrane, and eicosanoids [29]. The role of these lipid-mediators in heart physiopathology is supported by several recent studies [30,31,32]. In addition, the anti-inflammatory and pro-resolving effects of lipid mediators derived from ω-3 fatty acids were shown to inhibit atherosclerosis in clinical trials [33]. However, even if some lipidomic analyses have been published [22,34], the function of such signaling lipid cargo within the extracellular vesicles remain poorly described.

One work recently reported on an analysis of resolvins (Rv) and prostaglandins (PG) in MSC EVs [35]. Recently, the complete profiles of eicosanoids transported within the extracellular vesicles of rat BM-MSC and of cardiomyoblasts (H9c2) have been characterized [36]. The impact of polyunsaturated fatty acids (PUFAs) at physiological concentrations on these profiles has also been studied. PUFAs are known to be precursors of inflammatory signaling molecules and could support the almost resolving role that stromal cell-derived extracellular vesicles can have [36].

In the present work, we extend this study to mouse macrophages (J774) and to rat cardiac MSCs, opening up the landscape of the cardiac microenvironment to stromal cells described as orchestrators of inflammation and resolution processes. The aim remains to enhance our capacity to understand the paracrine exchange between the cardiac inflammation/resolution orchestrators. As it was only reported a few times that some enzymes encoding for eicosanoid synthesis could be transported by the EVs, we prove here that these are present in our models and that they are functional during their EV transportation. Moreover, we report the singular behavior of the EVs when secreted by J774 cells.

## 2. Results

### 2.1. EVs Characterization

Following the MISEV2018 [37], we performed three different physicochemical characterizations: nanoparticle tracking analysis (NTA), transmission electron micrography (TEM) and size exclusion chromatography (SEC) with BiCinchoninic acid Assay (BCA) Protein and NTA detections.

The cells (cMSC, H9c2 and J774) were plated at 5000 to 10,000 cells/cm^2^. When sub-confluence was reached, the cells were deprived, each over 24 h, to induce the same stress as those described in the literature. Then, the supernatants were collected for 24 h more. The apoptotic bodies (AB) were removed via a 10,000× *g* centrifugation. The EVs were collected after ultra-centrifugation at 100,000× *g* (1:30 h), suspended in PBS and submitted to NTA analysis.

**Figure 1:**
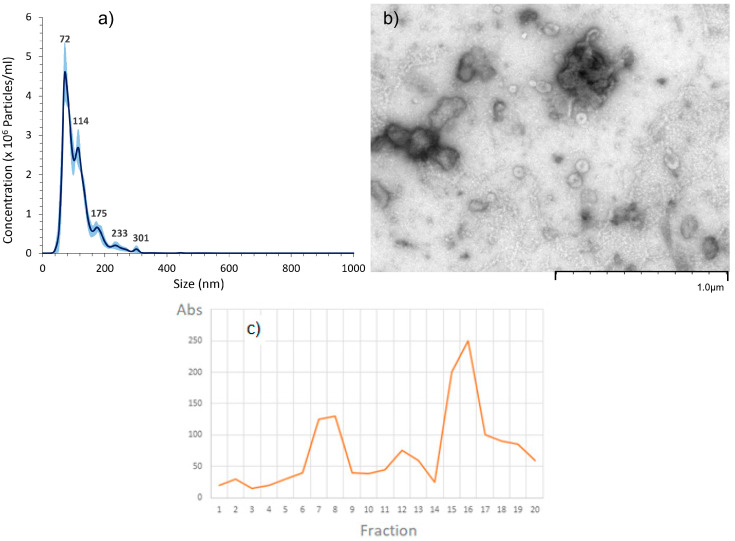
(**a**) Nanoparticle tracking analysis (NTA) and (**b**) transmission electron micrograph (TEM) of extracellular vesicles obtained after SEC on cMSCs (fraction 7–8) (**c**) qEV35 SEC separation with BCA protein quantification (absorbance at 570 nm).

We also performed size exclusion chromatography on an aqEV35 500 µL column. In accordance with the recommended protocol, we collected ten 0.5 mL fractions (Figure 1c) and measured the protein content using the BCA colorimetric method. The profile obtained is consistent with the presence of two populations of EVs. The first eluting in fraction 7 (proposed by the manufacturer Izon as containing the nanovesicles), and the other around fraction 9.

**Figure 2:**
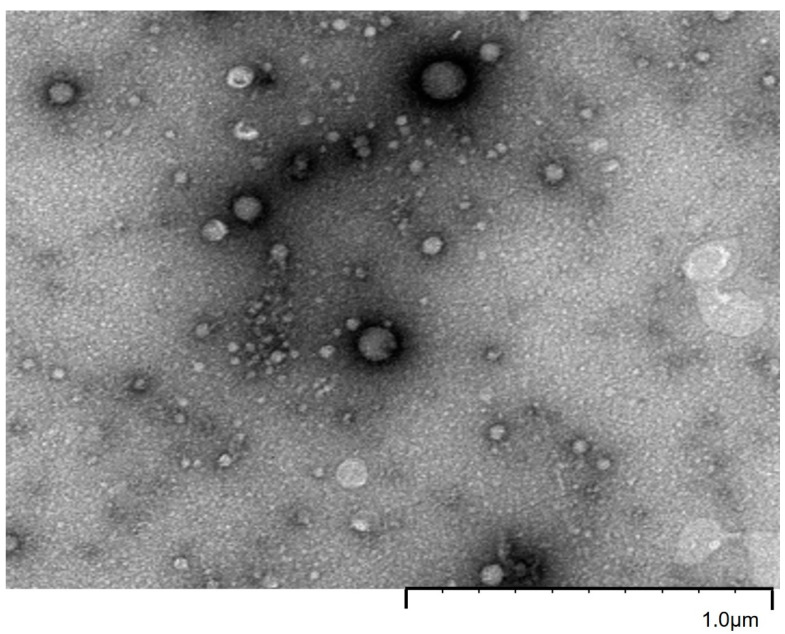
Transmission electron micrograph of EVs obtained from the crude fraction of cMSCs.

First, the crude fraction of cMSCs, suspended in PBS, was analyzed using transmission electron microscopy (TEM). A large population of spherical particles ranging from 30 to 100 nm was observed (Figure 2), corresponding to extracellular vesicles, as previously described with cryo-TEM [36]. Very few larger non-spherical objects were detected. These were most probably residual ABs.

The sample was then purified using the qEV35 size exclusion cartridge (Izon).

To determine the size distribution of the fractions supposed to enclose EVs, NTA was used as it provides accurate measurements even for polydisperse samples. Thus, no complementary measurements via dynamic light scattering were needed.

The size distribution obtained via the granulometric analysis performed with NTA showed that the sample was relatively polydisperse, with approximately 90% of the particles smaller than 170 nm. The size of the two main populations of particles were measured with NTA, centered on 72 nm and 114 nm (Figure 1a). They correspond to the targeted EVs. The concentrations in particles, reduced to initial medium volume were, respectively, at 4.5 × 10^6^ and 2.8 × 10^6^ particles/mL. To confirm that the signals observed in NTA around 70 and 110 nm could be attributed to extracellular vesicles, TEM experiments were performed. Again, two main populations of particles were detected around 100 nm and a smaller one around 70 nm. These populations were composed of spherical vesicles, which can be attributed to EVs. As already observed by Sahlen [38] and Aalberts [39], these two populations most probably have different compositions as they exhibit different electron densities, as shown with the EVs in Figure 1b and Figure 2. The prevalence of the small vesicles at around 70 nm observed with NTA (Figure 1a) was confirmed with the over-representation of a less dense population observed in the TEM image (Figure 2). Purification with SEC did not significantly impact the size distribution compared to the crude fraction, indicating that the last is pure. Indeed, NTA and TEM performed on fraction 7–8 (Figure 1) and fraction 9 demonstrated that nanovesicles were present in high amounts, meaning that the protein peaks observed on the SEC (Figure 1c) were not due to protein-aggregates.

In order to validate that the particles obtained from deprived J774 cell cultures could also be attributed to EVs, the same experiments were performed.

**Figure 3:**
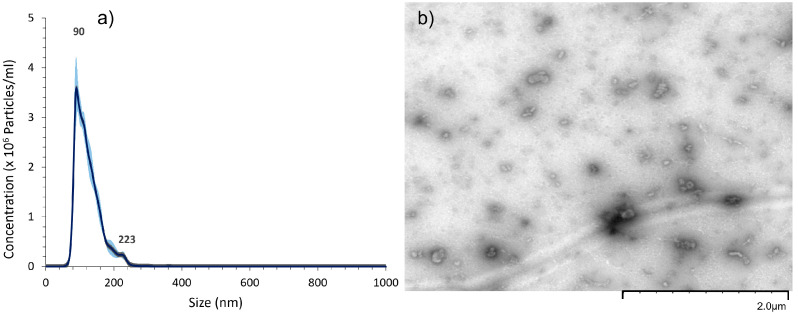
(**a**) Nanoparticle tracking analysis (NTA) and (**b**) transmission electron micrograph (TEM) of extracellular vesicles obtained from mouse macrophage J774 cultures.

Similar results were obtained from granulometric analysis. A total of 90% of particles were smaller than 170 nm, with a main population centered on 90 nm at a concentration of 3.5 × 10^6^ particles/mL (Figure 3a) after a ×100 dilution. The TEM image also shows small spherical objects of about 100 nm, validating the presence of extracellular vesicles. Notably, the number of particles secreted by the mouse monocytes were in the same range as the one secreted by the MSCs, described as performant secretors [11].

To confirm the significant presence of exosomes within these nanometric spherical objects, immunodetection of Alix, an exosome-specific marker, and of HS60, a cell-specific marker, were performed on these small EVs fractions (see Section 2.5).

### 2.2. Small EVs from J774 Macrophages Carry Lipid Mediator

Similar to the studies conducted in our 2020 publication [36], we wondered if we could analyze the lipid mediators released by small phagocytic cells such as macrophages. Since cardiac macrophages are difficult to maintain in culture, we opted for the immortalized commercial model J774. After a 24 h deprivation and 24 h of extracellular vesicle collection, we determined that the amount of small EVs produced by J774 was comparable to the ones produced by the cMSCs. The analysis of the free lipids transported by the small EVs, indicated that, as observed for the other cells we studied, the eicosanoid profile is dominated by two types of hydroxylated compounds: hydroxyeicosatetraenoic acids (HETE) and lipoxins (LX) (Figure 3).

**Figure 4:**
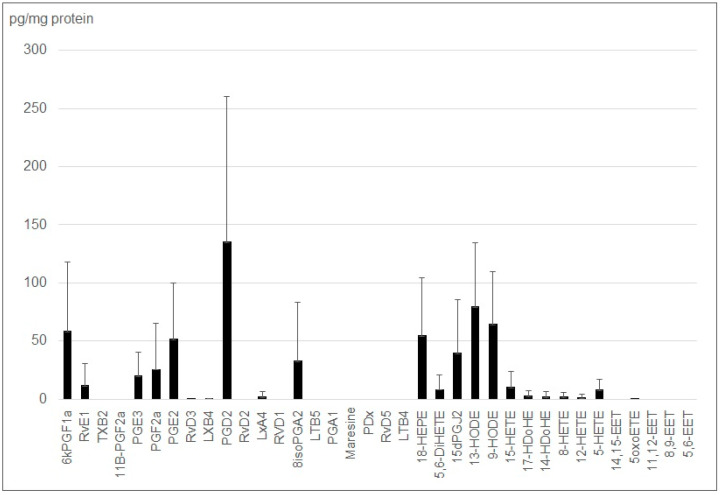
Lipid mediators’ profile from small EVs obtained from deprived J774 cell cultures in triplicate.

It should be noted that even if the eicosanoid profiles are similar from one replicate to the other, the low free lipid content, often close to the detection limit for the J774 EVs, makes their quantification difficult. This is almost linked to their low EV production level (compared to the MSCs or myoblasts), resulting in low eicosanoid concentrations, a problem that is amplified by the heavy analysis protocol, as indicated by the error bars. In particular, significant amounts of resolvins (RvE1) could be systematically characterized, but are very close to the quantification limit. However, we found that, unlike the MSC and H9c2, the content of lipid mediators transported within the small EVs is reduced (compared to the quantity of cellular proteins) by a factor of 10 and their profile is close to the one of the parent cells (Figure 4).

### 2.3. The Cargo of Small EV’s from J774 Macrophages Is Sensitive to PUFA

**Figure 5:**
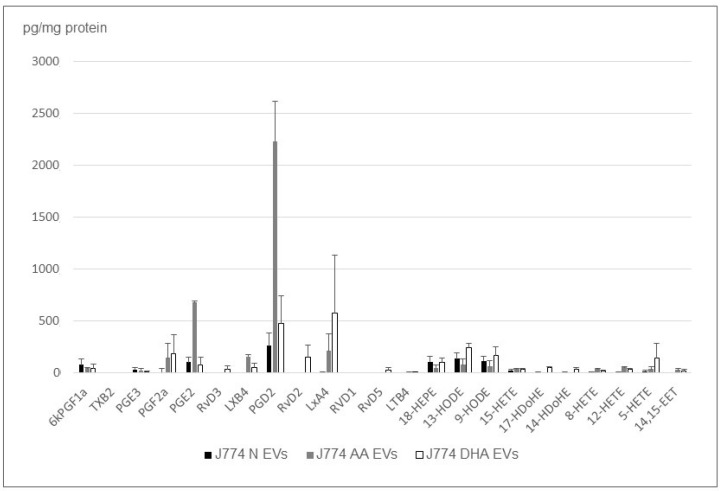
Lipid mediator profile from EVs obtained from deprived J774 cell cultured in triplicate without long chain fatty acid supplementation (N), or supplemented, respectively, with 10 µM of arachidonic acid (AA), eicosapentaenoic acid (EPA) or docosahexaenoic acid (DHA).

Similar to the studies we conducted in 2020 [36], we investigated the impact of the lipid sources introduced into the cell culture medium on the J774 EV lipid cargo. We deprived the J774 cells with 1% ultra-centrifuged bovine fetal serum, and supplemented them afterwards with 10 µM of the n-6 PUFAs: arachidonic acid (AA) and the n-3, eicosapentaenoic acid (EPA) or docosahexaenoic acid (DHA). As expected, some compounds, directly linked to the presence of their precursor introduced into the culture medium, were amplified (PGs, hydroxyeicosapentaenoic acid (HEPE) or RvD, respectively, to AA, EPA, DHA). Nevertheless, in contrast to the major modifications we published previously [36], the complete profile of lipid mediators secreted by this macrophage lineage remains globally similar before and after the addition of PUFAs (Figure 5).

### 2.4. Small EV Cargo of Rat Cardiac MSC and Mouse Monocytes Changes when Cells Are Treated with PUFAs

In order to understand if the amplification of the profiles in the case of the J774 macrophages or of the cardiac MSC’s is due to a specific enrichment piloted by the cell or by the small EVs themselves, we incubated the EVs collected from normal cell cultures within media supplemented with the n-6 and n-3 PUFAs. Thanks to the high EV production level by cMSC, the eicosanoid quantification was more robust, as indicated by the error bars.

Cardiac heart MSCs were identified based on CD45−, CD31−, Thy1+, CD44+, CD29+ and CD73+ expression after extraction from adult rat hearts and selection via adhesion on culture dishes, as stated in the Materials and Methods section.

**Figure 6:**
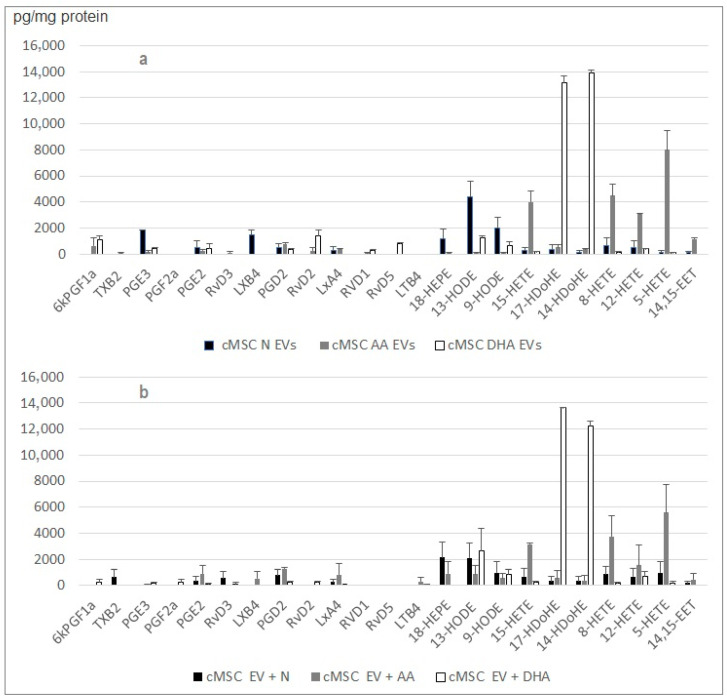
Comparison between the lipid mediator profiles embedded in (**a**) small EVs released by cMSC cultured in the presence of PUFAs and (**b**) small EVs released by cMSC cultured in MEMα 1% FBS that were incubated after collection in MEMα supplemented with PUFAs. No lipid mediators were detected in the absence of small EVs. Number or replicate was four.

The lipidomic analyses indicated that the small EVs produced by cMSC cells can synthesize identical amounts and compositions of lipid mediators to the small EVs released by the treated cells (Figure 6).

**Figure 7:**
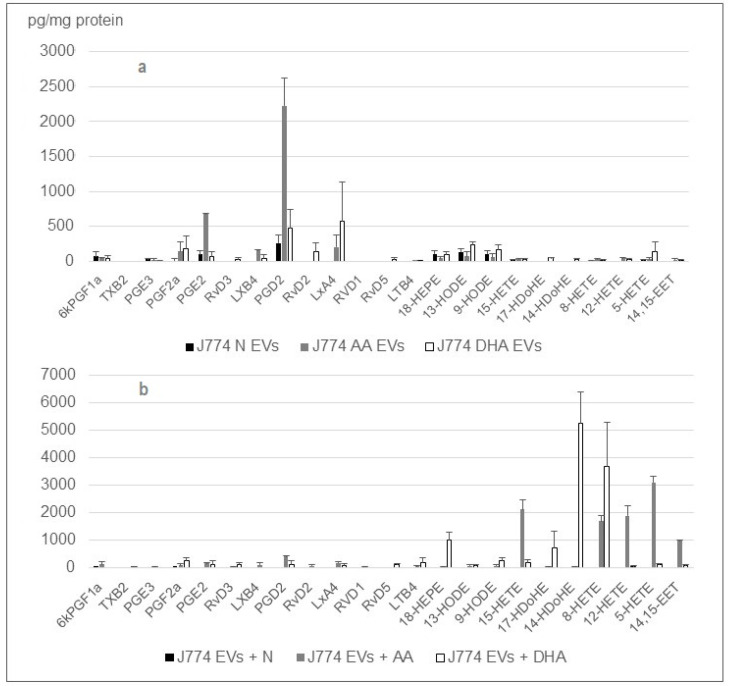
Comparison between the lipid mediator profiles embedded in (**a**) small EVs released by J774 cultured in the presence of PUFAs and (**b**) small EVs released by J774 cultured in DMEM 1% FBS that were incubated after collection in DMEM supplemented with PUFAs. Number or replicate was three.

The J774 behavior is very different from the ones of the cardiac stroma cells (Figure 7). The lipid mediator profiles embedded in small EVs released by J774 cultured in the presence of PUFAs are poor. Contrarily to the MSCs, the embedded hydroxyacids are not enriched, and therefore, the prostaglandins remain quite dominant. However, the lipid mediator profiles embedded in small EVs (released by J774 cultured in DMEM 1%) that have been incubated in DMEM supplemented with PUFAs, exhibit the attempted profiles (high HETE levels), indicating that the functional lipoxygenases (LO) are present in the EV cargo.

### 2.5. Small EVs from the Cardiac Stroma Carry Functional Enzymes to Synthesize Lipid Mediators

To verify that the observed autonomous biosynthesis by the EVs is effective, we investigated the presence of the biosynthetic enzymes within the EVs. We chose to investigate the enzymes synthesized by the MSC from rat hearts and the J774 mouse monocytes. We also added H9c2 rat cardiomyoblasts (described in [36]) on the blots. Indeed, we published at this time the activation of some RNAs involved in the eicosanoid synthesis but did not demonstrate the presence of their products. We collected small EVs from nine large Petri dishes and submitted them to Western blot analyses with the specific detection of the antibodies targeting the aimed synthases. As a consequence of the lipidomic results, we focused on 12-, 15- and 5-LO for the hydroxylated eicosanoid synthesis, as well as on PGE2 and PGD2 (especially for the H9c2 background) synthases. The quality and purity of the EV fractions were validated by the presence of significant amounts of Alix (EV specific surface protein) and by the absence of hsp60, a protein specifically expressed on mitochondria, a cellular organelle with the size of nanovesicles. A positive antibody-labelling reaction was assessed using tissues known to be rich in the targeted enzyme (heart for the prostaglandin synthases PGES and PGDS, lung for 5-lipoxygenase (5-LO) and spleen for 15-lipoxygenase (15-LO)).

**Figure 8:**
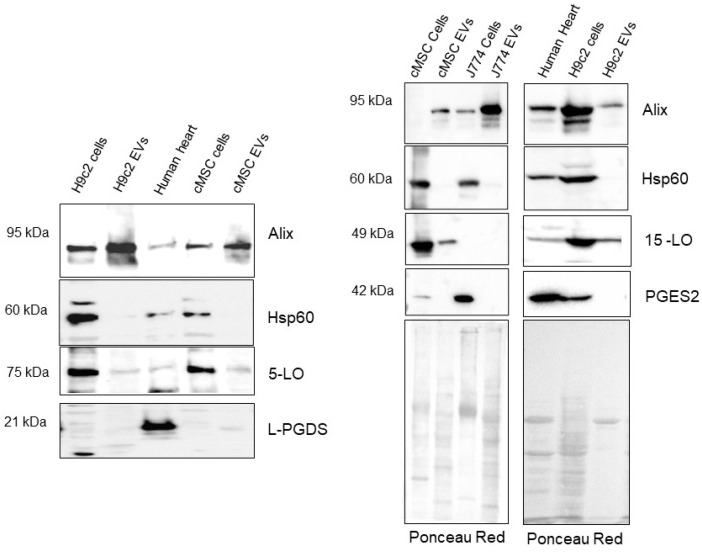
Immunoblots performed on the cells and small EVs produced by the rat cardiac cells (H9c2 and cMSC) and the J774 monocytes. MW: size markers. Different EV productions were used for each blot. For each blot, the upper panels correspond to the antibody recognition and the global protein distribution at the bottom. Positive recognition was performed with human heart tissue.

PGES2, 15-LO and 5-LO were present in the investigated cells (cMSC, H9c2), but only PGES2 and 5-LO were found in J774 cells. The 5-LO and 15-LO are the only enzymes we could demonstrate as being embedded within the small EVs (Figure 8).

Even if the prostaglandin PGD2 is often enriched in the EVs and PGES2 was abundant in cells studied, no PGD2 synthase signal could be observed in small EVs. We were not able to characterize the PGD2 synthase expressed in cMSC and H9c2. Unfortunately, hematopoietic PGDS antibody could not be tested here.

## 3. Discussion

Our previously published work demonstrated that small EVs produced by cardiac fibroblasts (cF), MSCs and H9c2 were enriched in lipoxin B4 (LXB4), PGD2 and HETEs compared to their parent cells [36]. After the incubation of cells with the ω-3 and -6 PUFAs (EPA, DHA and AA), the PUFA-derived metabolites were transferred into the small EVs in a specific manner. Besides this observation, we found that the small EVs produced by cardiac fibroblasts and MSCs were far more efficient in carrying the eicosanoids than the ones released by the cardiomyoblasts H9c2. We hypothesized that these changes in the oxilipin profiles could impact the inflammation resolution, as the enriched oxilipins could be qualified as being more anti-inflammatory compared to the parent cells, even if only few resolvins could be observed.

These observations raised several questions we wished to address: Are the small EVs specific lipid-mediator-signaling vehicles? Are the small EVs simple carriers, or active signaling bodies? Is it mesenchymal cell specific, or are other circulating stroma cells as efficient?

Formerly, we showed that, even if the oxylipin profiles of cardiac MSCs obtained from adult rat hearts are not exactly like the ones observed in bone marrow, the differences reported between the cell content and the small EV content remain comparable. Surprisingly, in the present work, the lipid mediator profile embedded in the small EVs released by J774 cultured in standard conditions (DMEM 1% bovine fetal serum) resembles those produced by the cells, even if their amount was reduced. Moreover, when cultured under PUFA supplementation, J774 cells do not transferring the lipid mediator profiles into the released small EVs. This observation would indicate that in the J774 background, small EVs do not embed specific eicosanoid signals, but carry the lipids present in the parent cytosol.

However, when the small EVs produced by the J774 cells cultured under standard conditions are exposed to PUFAs (AA, EPA, DHA), the lipid mediator profile embedded changes radically. Huge amounts of eicosanoids and docosanoids are produced, especially HETEs and lipoxins, but also some resolvins. For the latter, even if they are close to the detection limit, they are systematically observed. This observation upheld, this time, the hypothesis that small EVs carry functional oxylipin biosynthetic machinery.

To assess this theory, we generated enough small EVs produced by cMSC, H9c2 and J774 cells under normal conditions to perform immunoblots on their cargo. Deduced from the lipidomic studies, we supposed that at least lipoxygenases might be globally present in the small EV cargo and possibly also PGD synthase in the H9c2 background. We assessed that the protein amounts were sufficient using Ponceau red coloration. Moreover, the efficiency of the antibody bindings was evaluated using specific tissues. Considering all these precautions, we could demonstrate that 5-LO and 15-LO were present in the small EVs secreted by the here-studied stromal cell. Contrarily to the suggestion made in the literature concerning the existence of a selective mechanism to load specific proteins onto EVs [40], the enzymes packed in the here-studied EVs are almost the same as those observed in their parent cell.

Other reports have shown that exosomecargoes are loaded with enzymes known to synthesize bioactive lipids. For example, exosomes secreted by hypoxic pulmonary artery endothelial cells are enriched in 15-LO2, whereas exosomes isolated from lung cancer pleural exudates or cancer cells contain enzymes capable of avidly converting LTC4 to the pro-tumorigenic cysteinyl leukotrien D4 [41,42]. However, it is, to our knowledge, the first report demonstrating that these enzymes are fully functional during their EV transportation and that the EVs allow molecular exchange out of the parent or target cells.

## 4. Materials and Methods

Due to its complexity, the protocol was schematized in Supplementary Figure S1.

### 4.1. Biological Material and Cell Cultures

The experiments were carried out on three cell types: a commercial H9c2 cardiomyoblast line (ATCC CRL-1446™, Sigma Aldrich, St Quentin-Fallavier, France), J774 monocyte/macrophage cell line and mesenchymal stem cells from adult Lewis rat hearts, which were obtained using a protocol previously described [43]. Briefly, hearts were harvested from rats after intraventricular perfusion of 20 mL PBS, minced with scalpels and digested with Liberase TM (Roche, Sigma Aldrich, St Quentin-Fallavier, France) diluted in RPMI1640 (GIBCO, ThemoFisher Scientific, Montpellier, France). The digestion of tissue fragments was performed via two successive incubations for 10 min with an enzymatic solution at 37 °C under shaking, and stopped by adding heat-inactivated fetal calf serum (HIFCS) (SIGMA). Single-cell suspensions were obtained via filtration on 100 µm cell strainers (Becton Dickinson Biosciences Discovery Labware, Le Pont de Claix, France). Red blood cells were removed via hypotonic shock with an ammonium chloride solution (0.83%). Cell suspensions were collected in αMEM (GIBCO) supplemented with 10% HIFCS and 1% Penicillin/Streptomycin and incubated for 24 h on a plastic dish for adhesion selection overnight. Non-adherent cells were removed, and the purity of cardiac MSC cultures was assessed via flow cytometry using CD29 PerCP (clone eBIOHMb1-1), CD45 PE clone OX-1, 0.7 µg/mL, CD90/Thy1 Bv421 (clone OX-7) from Biolegend or CD73 (clone 5F/B9 from Becton Dickinson Biosciences Discovery Labware, Le Pont de Claix, France, or CD31Vio^®^ Bright FITC, clone REA396, CD44H APC clone REA505) from Miltenyi biotech, Paris, France. Cells were incubated for 30 min with antibody diluted at 0.5 to 0.6 µg/mL in the dark on ice, and then the cells were washed with FACS buffer (PBS, 1% HIFCS, 0.5 mM EDTA). Then, dead cells were determined using a LIVE/DEAD Viability kit (ThemoFisher Scientific, Montpellier, France) (Supplementary Figure S2). The cell cultures were carried out in a CO_2_ incubator, thermostatically controlled at 37 °C, with 5% CO_2_. Cardiomyoblasts and J774 cells were cultured in Dulbecco’s Modified Eagle’s Medium (DMEM, ATCC, Sigma Aldrich, St Quentin-Fallavier, France) supplemented with 10% HIFCS and 1% Penicillin/Streptomycin culture medium. To collect the small EV fraction, the dishes were rinsed with 10 mL of PBS, and the medium was replaced with the same medium but deprived in 1% of ultracentrifuged fetal serum (2 h at 100,000× *g* to deplete it from its EVs) for 24 h. The supernatant was collected over an additional 24 h period after the renewal of the medium.

### 4.2. Protein Assay and Western Blotting

Protein extraction of the samples was carried out using the RIPA lysis buffer containing phosphatase and protease inhibitors (composition: TrisHCl 50 mM; NaCl 150 mM; NP40 1%; Desoxycholic Acid 1%; SDS 0, 1%; EDTA 5 mM; NaF 20 mM). The lysis was carried out in ice; 5 μL of the buffer was added to the samples, shaken with a vortex and finally incubated for 30 min at 4 °C. Total proteins were determined via the BCA method (BiCinchoninic acid Assay, Pierce, ThemoFisher Scientific, Montpellier, France) in a 96-well plate. A standard range was produced from a protein solution of BSA (bovine serum albumin, Sigma Aldrich, St Quentin-Fallavier, France) at 1 mg/mL (0; 62.5; 125; 250; 500; 1000 μg/mL). The samples were diluted 1/10 in PBS, and then 10 μL was deposited in the wells in the presence of 200 μL of the BCA reagent. After incubation at 37 °C for 30 min, the absorbance was measured at 570 nm using a spectrophotometer (TECAN).

Immunodetection was realized using Western blotting with 25 to 40 μg of purified extracellular vesicles fraction or cell suspension. Detection of oxylipin-synthesizing enzymes was performed using antibodies from Santa Cruz biotechnologies (5-LO (sc-136195), 15-LO2 (sc-271290), PGES2 (sc-514224); L-PGDS (sc-390717) and HPGDS (Genetex)). Hsp60 (sc-13115) and Alix (cell signaling) were used according to the manufacturer’s instructions. The positive signals were detected via chemiluminescence using Biorad clarity ECL substrate and analyzed using Image lab (Biorad, Marnes-la-Coquette, France).

### 4.3. Sample Preparation

The supernatants of the cell culture were centrifuged for 20 min at 10,000× *g* to remove the apoptotic bodies. The supernatants were then subjected to precipitation via ultracentrifugation (100,000× *g*; 1 h 30, 4 °C) (Beckman and Coulter).

Cells (around 4–5 × 10^6^ cMSC and 6–8 × 10^6^ J774/Petri dish) were collected using a scraper in 1 mL PBS and transferred to hemolysis tubes. An aliquot of 50 μL was taken to carry out an assay of the cellular proteins.

### 4.4. Small EV Characterization

#### 4.4.1. Nanoparticle Tracking Analysis

NTA analyses were performed at 25 °C using a NanoSight LM10 (Malvern Instruments, Ltd., Malvern, UK) instrument equipped with an sCMOS camera. The camera level was fixed at 13 with a detection threshold of 5. Samples were prepared via EV dispersion in 200 µL of PBS (GIBCO Phosphate Buffer Saline, without CaCl_2_ and MgCl_2_) and then diluted ×100 in ultrapure Milli-Q water, filtered at 1.2 µm. The measurement of each sample was performed in triplicate, each for 60 s. The concentration of particles was presented as a function of the mean size of the particles.

#### 4.4.2. Transmission Electron Microscopy

The presence of EVs was observed through the transmission electron microscopy (TEM) technique using a HT7700 Hitachi (Fukuoka, Japan) microscope operated at an accelerating voltage of 80 kV. Samples for TEM were prepared using the negative-staining method with 2% (*w*/*w*) uranyl acetate in water. Aliquots (~100 μL) of EV solution prepared in PBS were put onto copper glow discharged TEM grids for 1 min. Then, the grids were placed in the solution of uranyl acetate for another minute. Then, the grids were dried at room temperature before the analysis.

#### 4.4.3. qEV Size Exclusion Chromatography (Izon)

The pellets obtained from ultracentrifugation of the supernatant of 6 big Petri dishes of cMSC or J774 macrophages, cultured in MEM or DMEM, supplemented with 1% ultracentrifuged fetal serum, were pooled together and resuspended in 100 µL of PBS. They were fractionated on a 500 µL qEV35 size exclusion cartridge and eluted with PBS following the cartridge user manual. Shortly after, the cartridge was washed, the 100 µL crude fraction was deposited on the top, and 12 fractions of 500 µL were collected. BCA quantification (see Section 4.4) was performed on 20 µL of each fraction.

### 4.5. Free-Lipid Extraction

Deuterium-labeled eicosanoids (LxA4-d5, LTB4-d4, and 5-HETE-d8) were mixed at a 400 ng/mL concentration in methanol and used as an internal standard (IS) solution. The 1 mL cell and extracellular vesicle suspensions were placed in the presence of 10 μL of IS, and the lipid extraction was carried out twice in glass hemolysis tubes with 500 μL of chloroform. The samples were vortexed and left to settle. The organic phases were collected, mixed and evaporated to dryness under nitrogen flow at 45 °C. The samples were first stored in pill boxes with glass inserts in 20 μL of methanol at −80 °C, and then subjected to spectrometric analysis by LC/MS-MS.

### 4.6. HPLC/MS-MS Analysis

High-performance liquid chromatography was performed using an Agilent 1290 Infinity. The analytical column was a ZorbaxSB-C18 column (2.1 mm, 50 mm, 1.8 μm) (Agilent Technologies) maintained at 40 °C. The mobile phases consisted of water, acetonitrile, and formic acid (75:25:0.1; *v*/*v*/*v*) (A) or (0:100:0.1, *v*/*v*) (B). The linear gradient was as follows: 0% B at 0 min, 85% B at 8.5 min, 100% B at 9.5 min, 100% B at 10.5 min, and 0% B at 12 min. The flow rate was 0.35 mL/min and the injection volume was 5 μL. The HPLC system was coupled on-line to an Agilent 6460 triple quadrupole MS (Agilent Technologies). Electrospray ionization was performed in negative ion mode. Analyses were performed in selected reaction monitoring detection mode. Peak detection, integration, and quantitative analysis were conducted using Mass Hunter Quantitative analysis software (Agilent Technologies) via correlation with the IS response.

## 5. Conclusions

We report about three new insights. First, we highlighted that, in contrast to other cardiac stromal cells, the exposure of J774 (immortalized macrophages) to polyunsaturated fatty acid does not drastically impact the eicosanoid content of secreted small EVs. Secondly, we show that stromal cells’ small EVs (including for J774) carry the enzymatic machinery, allowing them to synthesize inflammation bioactive compounds. Finally, we report about the fact that this machinery is fully functional, as the small EVs, alone, produce high eicosanoid amounts by sensing the PUFAs in their environment.

The macrophage-specific behavior that we reveal indicates that small EVs can synthesize bioactive compounds independently, even when the EVs produced by the parent cells exposed to the same environment do not. Therefore, although the molecules found in EV cargo are constrained by their parental cells, these small vesicles carry the molecular machinery allowing the modulated expression of the desired molecular profile after secretion. This observation upheld the hypothesis that they are key factors for the paracrine signaling, even in absence of the parent cell. Moreover, it opens their potential circulating monitoring role. This hypothesis now must be explored, integrating the ability of these nanovesicles to deliver their cargo in cells or even in the surroundings, as was proposed recently for tongue cancer, where many deregulated EV proteins are found in the plasma from patients [44].

Indeed, it has yet to be discovered whether circulating or tissue EVs may have such autonomous synthesis function in vivo, or in interstitial fluid, or whether EVs are only used to transfer enzymatic material from cell to cell. Moreover, further investigations will be required to establish whether small-EV-derived cargos have functional significance in recipient cells as demonstrated by the delivery of EV-encapsulated RNA.

## Data Availability

The authors declare unshared data.

## Supplementary Materials

The supporting information can be downloaded at: https://www.mdpi.com/article/10.3390/ijms24065866/s1.
