# Supplementary figures and images for: Extracellular Vesicles Produced by the Cardiac Microenvironment Carry Functional Enzymes to Produce Lipid Mediators In Situ
Source: Int J Mol Sci. 2023 Mar 20;24(6):5866. doi: 10.3390/ijms24065866 (PMC10056942; doi:10.3390/ijms24065866)

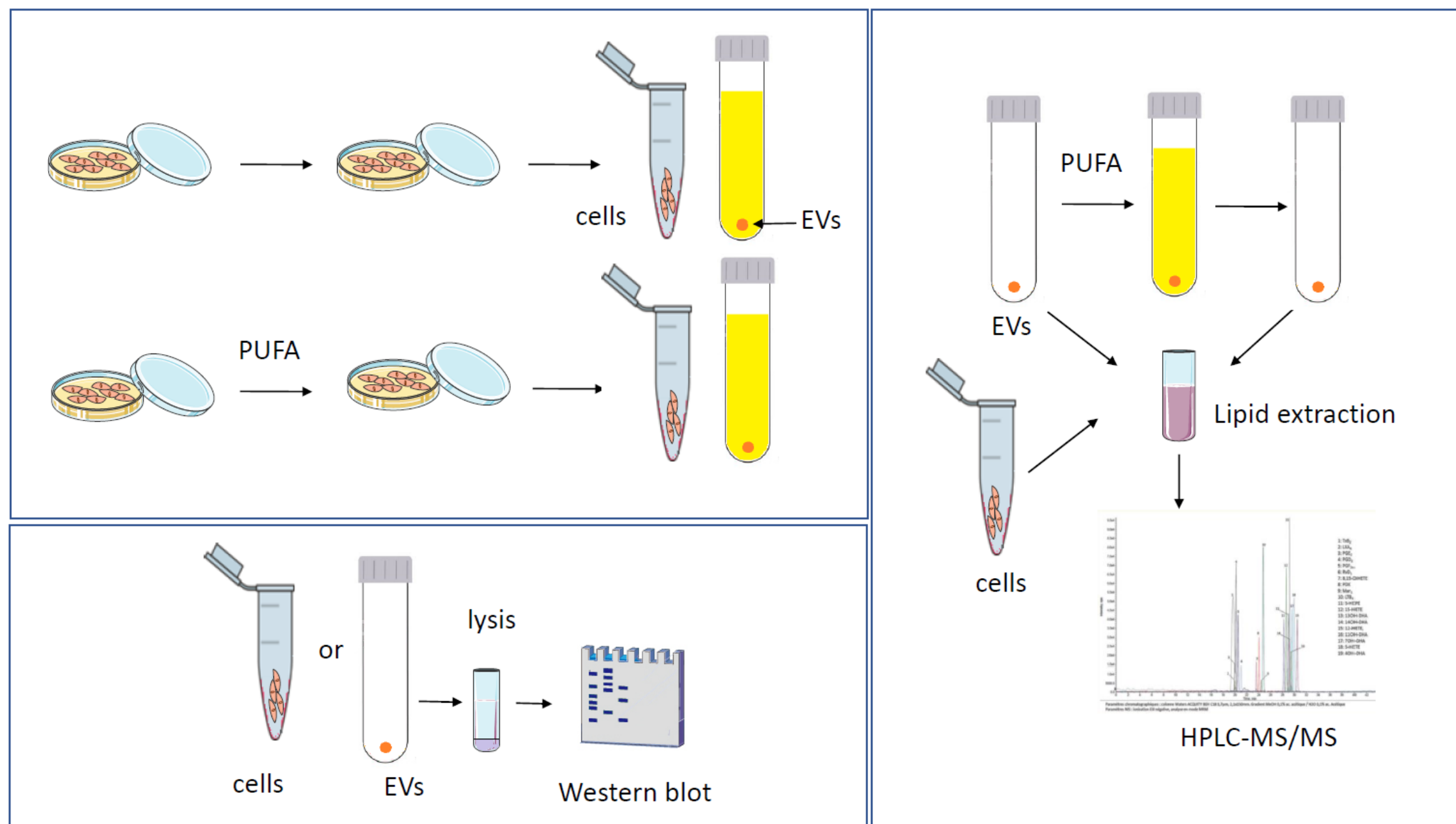

Figure S1

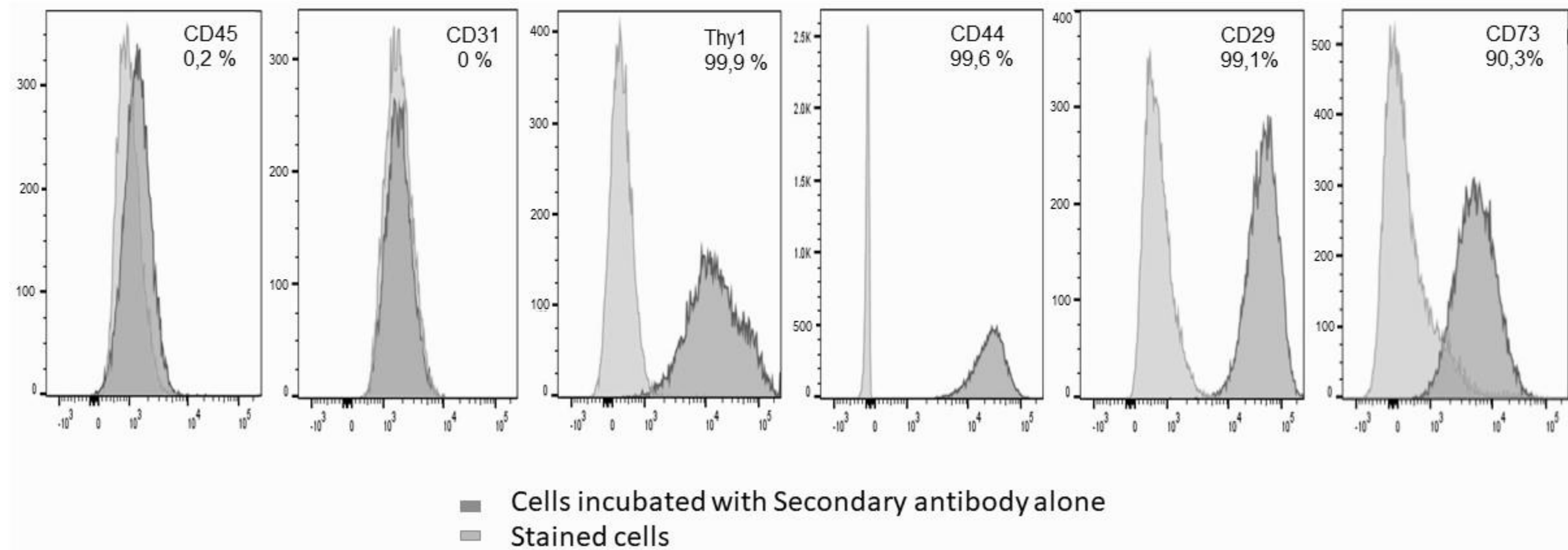

Figure S2

Supplement: Supplementary file 1 [file ijms-24-05866-s001.zip › ijms-2221302-supplementary.pdf]
